# Determinants of healthy lifestyle behaviours in colorectal cancer survivors: a systematic review

**DOI:** 10.1007/s00520-025-09315-x

**Published:** 2025-03-18

**Authors:** Judith de Vries-ten Have, Renate M. Winkels, Sharon A. G. Bloemhof, Annelot Zondervan, Iris Krabbenborg, Ellen Kampman, Laura H. H. Winkens

**Affiliations:** 1 Nutrition & Disease Chair Group, Division of Human Nutrition and Health, Wageningen University & Research, Wageningen, The Netherlands; 2 Consumption and Healthy Lifestyles Chair Group, Wageningen University & Research, Wageningen, The Netherlands

**Keywords:** Behaviour change, Diet, Exercise, Lifestyle, Physical activity

## Abstract

**Purpose:**

Identifying and selecting determinants of health behaviours is an important step in the design of behaviour change interventions. Many colorectal cancer (CRC) survivors experience disease- and treatment-related complaints, which may make it difficult to implement behavioural changes. In this systematic review, we aimed to identify determinants of a healthy lifestyle, i.e. dietary behaviours and physical activity, in CRC survivors who finished treatment.

**Methods:**

We searched Web of Science, PubMed and PsychINFO, to retrieve quantitative and qualitative studies on determinants of a healthy lifestyle in CRC survivors who finished treatment. Synonyms of the following search terms were used: ‘CRC survivors’, ‘lifestyle’, ‘physical activity’, ‘nutrition’ and ‘determinant’. The level of evidence for each determinant was classified as ‘convincing’, ‘moderately convincing’ or ‘unconvincing’ based on consistency of findings between studies and quality of studies assessed with the Mixed Methods Appraisal tool.

**Results:**

Twenty-one studies were retrieved of which twenty were classified as ‘high-quality studies’ and one as ‘low-quality study’. Determinants that were convincingly associated with less healthy lifestyle behaviours were smoking, depression, body image distress/consciousness, experiencing pain, dealing with symptoms and bad health status. A good functional status was convincingly associated with more healthy lifestyle behaviours. Determinants with convincing evidence for an association with less or more healthy lifestyle behaviours were time and other priorities, knowledge, motivation, (false) beliefs, perceived and expected outcomes, skills, social support, social norms and influence, access to facilities and equipment and weather.

**Conclusion:**

Interventions for changing health behaviours in CRC survivors who finished treatment could use these determinants to tailor and personalize the intervention to the target group.

**Supplementary Information:**

The online version contains supplementary material available at 10.1007/s00520-025-09315-x.

## Introduction

There are indications that healthy lifestyle behaviours, specifically healthy dietary behaviours and sufficient physical activity and/or exercise, are associated with lower all-cause mortality and reduced risk of recurrence in colorectal cancer (CRC) survivors [1, 2]. Additionally, healthy lifestyle behaviours have been associated with other health benefits, such as improved quality of life [3–5] and reduced cancer-related fatigue [4–7]. Regarding physical activity and dietary behaviours, the World Cancer Research Fund (WCRF) and American Institute for Cancer Research (AICR) generally recommend CRC survivors to follow the cancer prevention guidelines which involve staying physically active, including 150 min of moderate to vigorous physical activity per week, consuming a diet rich in vegetables, fruits, whole grains and beans, and limiting fast foods, red and processed meats, sugar sweetened beverages and alcohol intake [8, 9]. However, CRC survivors do not seem to change their lifestyle to a great extent in the years after diagnosis [10]. One of the reasons for not adopting a healthier lifestyle could be that many CRC survivors are experiencing disease- and treatment-related complaints, such as gastrointestinal problems, body image distress and cancer-related fatigue [11], which may make it difficult to implement behavioural changes.

Interventions that aim to change health behaviours, such as healthy eating and physical activity, are often complex interventions [12, 13]. These are interventions that contain several interacting components, involving a range of behaviours, expertise and skills; a number of groups, organisational levels or settings that are targeted; and a certain flexibility or tailoring of the intervention or its components [12, 13]. The use of a structured intervention development protocol can help to overcome the challenges of designing and implementing a complex intervention by establishing how the intervention works and evaluating which components contribute to the effectiveness of a trial [12]. An essential step in the design of a behaviour change intervention is the identification and selection of relevant determinants of that specific behaviour. Insight into which determinants are applicable in CRC survivors gives further direction to which factors should be targeted to adopt healthy lifestyle behaviours.

CRC survivors who completed treatment might have different factors influencing health behaviours than CRC survivors undergoing treatment. These factors include dealing with a (temporary) stoma and treatment-related side effects. Furthermore, the perspective of CRC survivors might shift from the focus on survival to dealing with the aftermath of the disease, such as the fear of recurrence [14, 15], dealing with physical symptoms and returning to work [15]. In systematic reviews, it is therefore important to clearly state where the CRC survivors are in their cancer journey. Systematic reviews on the determinants of healthy lifestyle behaviours for CRC survivors are sparse in the literature; only one overview could be found for CRC survivors, but this was focused solely on healthy eating behaviours and not physical activity [16]. In addition, that review did not differentiate the stage of their cancer journey [16]. Other studies [17–19] provided overviews of determinants for solely physical activity or exercise, and not nutrition, in adult cancer survivors, but did not focus specifically on CRC survivors. CRC survivors may have other determinants for healthy eating, physical activity and/or exercise than other cancer survivors. For example, a stoma and/or bowel dysfunction [20] may impact their eating behaviour and ability to engage in physical activity.

Therefore, this study aimed to systematically review the literature on determinants of healthy eating, physical activity and/or exercise in CRC survivors who completed treatment.

## Methods

The PRISMA statement was used for conducting the systematic review [21]. Various synonyms of the following search terms were used in search queries: ‘colorectal cancer survivors’, ‘lifestyle’, ‘physical activity’, ‘nutrition’ and ‘determinant’. The search query and eligibility criteria are displayed in Table 1. Three databases were queried: Web of Science, PubMed and PsychINFO. No filters or limits were used. The literature search was conducted from database commencement until March 2024. Trials, observational studies and qualitative studies were eligible. Rayyan® was used to manage records and data. Three authors conducted the initial search and deleted duplicates. Four authors screened the remaining abstracts and titles for eligibility independently. Full text screening was performed independently by four authors, with each record being checked by two authors. Conflicts were discussed and resolved with the review team.

**Table 1:** Search query used in the databases Web of Science, PubMed and PsychINFO and eligibility criteria for systematic review on determinants of healthy lifestyle behaviours in colorectal cancer survivors who completed treatment

Search query	Eligibility criteria
(“colorectal cancer survivors” OR “CRC survivors” OR “recovered colorectal cancer patients” OR “colorectal cancer survivorship” OR “colon cancer survivors” OR “recovered colon cancer patients” OR “colon cancer survivorship” OR “rectal cancer survivors” OR “recovered rectal cancer survivors” OR “rectal cancer survivorship”) AND (lifestyle OR exercise OR physical activity OR nutrition OR diet* OR consumption OR eating OR healthy eating OR dietary habits OR “dietary intake” OR “food intake” OR “health behaviours” OR “health perceptions” OR “dietary guidelines”) AND (“behavioural determinant” OR determinant OR barrier* OR facilitator* OR facilitating OR factor OR motivation OR predictor OR psychosocial OR correlate)	Inclusion:
	1. CRC survivors who completed all treatment
	2. Population > 16 years old
	3. Determinants in relation to physical activity and/or exercise, and/or diet are described
	4. Written in English
	5. Published in peer-reviewed journals
	Exclusion:
	1. Other cancer types
	2. Posters, conference abstracts, thesis dissertations and trial registries

### Data extraction and grouping of determinants

Data were extracted from the eligible full texts and was performed independently in duplicate by three authors. Conflicts were discussed and resolved within the review team. The following variables were extracted from the records, if provided: country, population, sample size, age, sex, stage of disease, time since treatment or diagnosis, study design, outcome (i.e. physical activity, exercise and/or nutrition-related), theoretical underpinning and determinants with results of statistical tests if applicable. Studies were divided into having a quantitative or qualitative approach towards identifying behavioural determinants and were analysed separately. For all studies, the description of the identified or analysed factors or theme were then used to group them into categories: socio-demographic factors, clinical factors, lifestyle factors, intrapersonal factors, social environment and interpersonal factors, (mental) well-being factors, disease-related symptoms and environmental factors. Certain studies discussed general barriers and/or facilitators, such as seasonal issues or the lack of social support, which we did not regard as independent determinants. Therefore, they were categorized under the most appropriate determinant group. The determinants in quantitative studies were classified as showing either positive, negative or no association based on statistical significance indicated by confidence intervals or *p*-values.

### Quality assessment

The quality of included studies was assessed with the Mixed Methods Appraisal tool, version 2018 [22]. This tool can be used to assess the quality of quantitative, qualitative and mixed methods studies. This involves a three-step process [22, 23]. First, an assessment is made if the study can be appraised at all, which is based on whether there are clear research questions and if the collected data allows to address these questions [22, 23]. Second, the appropriate category of study designs is determined from five categories: qualitative, quantitative randomized controlled trials, quantitative non-randomized, quantitative descriptive and mixed methods studies [22, 23]. Third, the study is rated on five criteria of the chosen study category with ‘Yes’, meaning criteria is met; ‘No’, meaning criteria is not met; and ‘Can’t tell’, meaning information is not convincing to judge this criterion [22, 23]. The quality assessment was performed by three authors independently, with each record being assessed by two authors, and conflicts were discussed and resolved within the review team. It is not advised by the developers of the tool to calculate an overall score of the quality [22, 23]. Therefore, the answer to each question per study can be found in Appendix Tables S1 and S2. However, to consider the quality of studies when assessing possible determinants, the studies were classified as ‘high-quality’ when ≥ 60% of questions could be answered with ‘Yes’. Low-quality studies were studies scoring ‘Yes’ on < 60% of questions.

### Classification of the level of evidence

For both quantitative and qualitative papers, we classified the level of evidence in line with a comparable systematic review on determinants of exercise in cancer survivors [18]. The level of evidence was classified as ‘convincing’ when there were consistent findings in ≥ 2 high-quality studies, ‘moderately convincing’ when findings were consistent in one high-quality study and at least one low-quality study or were consistent in ≥ 2 low-quality studies and ‘unconvincing’ when there was only one study available, or findings were inconsistent in ≥ 2 studies. Results were consistent when ≥ 75% of the studies showed results in the same direction [18]. To summarize the evidence, factors that had convincing evidence in either the quantitative studies or qualitative studies were considered determinants of healthy lifestyle behaviours.

## Results

### Characteristics of included studies

**Fig. 1:**
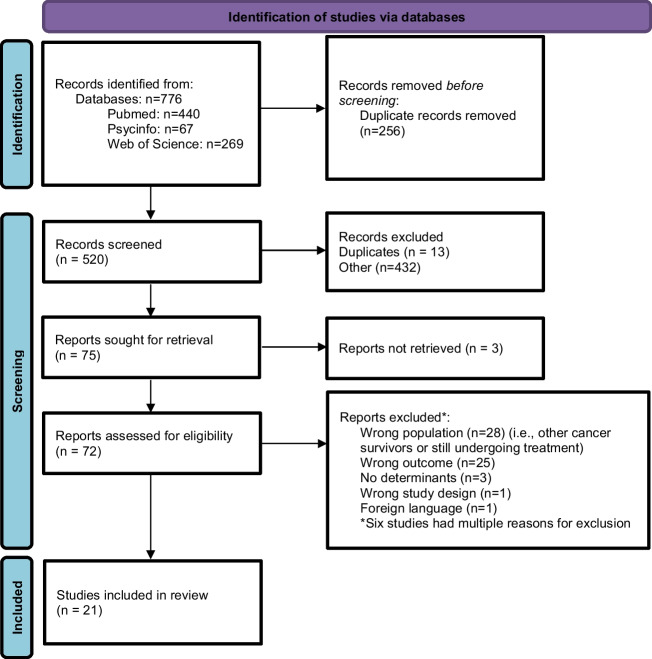
Study selection systematic review, figure adapted from Page et al. (2020) [21]

In total 776 studies were identified, of which 520 studies were reviewed for title and abstract screening after removing of duplicates (Fig. 1). Hereafter, 72 studies were screened for full text of which 21 studies were included [24–44]. The sample sizes of the studies ranged from 15 to 2451 participants with a median sample size of 96 participants (Table 2). Studies conducted interviews, focus groups, surveys and/or used accelerometer-based data; for details, see Table 2. Three studies examined study outcomes related to nutrition [34, 36, 38], 15 studies examined study outcomes related to physical activity or exercise [24–33, 35, 37, 39–41], and 3 studies examined outcomes related to both nutrition and physical activity or exercise [42–44]. Thirteen studies used behavioural theories or models as a backbone to conduct the studies or explain the data analysis [24–26, 28, 29, 31, 33, 35, 37–40, 42]. The most frequently used theory was the theory of planned behaviour, which was used in six studies [24, 26, 33, 35, 37, 42]. Twelve studies were classified as ‘quantitative descriptive studies’ [24–35] and nine studies as ‘qualitative studies’ [36–44] during the quality assessment. Twenty out of the 21 studies were classified as ‘high-quality studies’ [24–27, 29–44] and one study as ‘low-quality study’ [28], which was due to the sampling strategy not being relevant to the research question, inappropriate measurements and a high risk of non-response bias (Table 2, S1, S2).

**Table 2:** Characteristics of included studies (*n* = 21)

Study	Country	Population (sample, age, sex)	Disease (stage, time since treatment or diagnosis)	Study design	Physical activity / nutrition	Theoretical underpinning	Type and quality of the study^†^	Analysed as quantitative, qualitative or both study type^‡^
Bours et al. (2015) [34]	Nether-lands	1458 CRC survivors aged 70.2 ± 9 years, with 43% female	Stage I–IV 6.9 ± 3 years since diagnosis	Surveys	Dietary changes—11 food components and nutritional products	-	Quantitative descriptive 80%	Both
Speed-Andrews et al. (2014) [35]	Canada	600 CRC survivors aged 67.3 (range 31–92) years, with 41.7% female	Stage I–IV 51 months after diagnosis (range 8–514 months)	Self-reported questionnaires	Physical activity	Theory of Planned Behaviour	Quantitative descriptive 80%	Both
Hardcastle et al. (2018) [43]	Australia	24 CRC survivors aged 69.4 ± 4.2 range (63–77) years, with 54.2% female	Stage A–C Within 2 years since treatment	Semi-structured face-to-face interviews	Health concerns, diet and physical activity	-	Qualitative 100%	Qualitative
Hardcastle et al. (2017) [44]	Australia	24 CRC survivors aged 69.4 ± 4.2 range (63–77) years, with about half men (not specified)	Stage of disease was not specified Within 2 years since treatment	Semi-structured face-to-face interviews	Health behaviours (exercise and healthy eating)	-	Qualitative 100%	Qualitative
Harper et al. (2013) [42]	USA	17 older African American CRC survivors aged 74.1 ± 5.9 (range 58–79) years, with 47% female	Stage of disease was not specified 2–10 years after diagnosis	Semi-structured focus groups and telephone interviews	Health behaviours (e.g., diet and exercise)	Theory of Planned Behaviour, Bandura, culturally relevant beliefs	Qualitative 100%	Qualitative
Maxwell-Smith et al. (2017) [41]	Australia	24 CRC survivors at high risk of CVD aged 69.4 ± 4.2 (range 63–77) years, with 54.2% female	Stage of disease was not specified Within 2 years since treatment	Semi-structured interviews	Physical activity	-	Qualitative 100%	Qualitative
Ray et al. (2018) [40]	USA	30 African American and White CRC survivors aged 40–74 years, with 63% female	All stages Completed all active treatment	Semi-structured interviews	Exercise	PEN-3 model: perceptions, enablers, nurtures	Qualitative 100%	Qualitative
Saunders et al. (2019) [39]	Canada	15 stage rectal cancer survivors with a stoma aged 59.4 (range 34–80) years, with 26.7% female	Stage II–IV (2 survivors had unsure stage of disease) Completed surgery for stoma and chemotherapy	Semi-structured interviews	Engagement in physical activity	Ontological realism, epistemological contextualism	Qualitative 80%	Qualitative
Tang et al. (2019) [38]	China	30 CRC survivors aged 69.9 years, with 56.7% female	Stage 0–III Within 1 year of finishing surgery and any adjuvant therapies	Individual semi-structured interviews	Post-diagnosis dietary decision-making	Grounded theory	Qualitative 100%	Qualitative
Byeon et al. (2024) [37]	Korea	17 CRC survivors aged 39–67 (mean 55.9) years, with 58.8% female	Stage 0–3 2.2 years post-treatment	Semi-structured face-to-face interviews	Physical activity and exercise	Theory of Planned Behaviour	Qualitative 100%	Qualitative
Liu et al. (2023) [33]	USA	42 CRC survivors with median age 56 [interquartile range 48, 63] years, with 57% female	Stage I–IV 1 year [interquartile range 0,8] after diagnoses	Accelerometer-based assessments and survey	Exercise	Theory of Planned Behaviour	Quantitative descriptive 80%	Qualitative
Wong et al. (2021) [36]	China	55 CRC survivors aged 64.1 ± 10.0 years, with 47.3% female	Stage I–IV Time since treatment was not specified	Individual and focus group interviews	Dietary change	…	Qualitative 100%	Qualitative
Chambers et al. (2009) [32]	Australia	978 CRC survivors, with highest proportion of 37.2% aged 70 + years, with 44.2% female	Stage Dukes’ A–D 5–36 months after diagnosis	Survey and computer-assisted telephone interview	Physical activity	-	Quantitative descriptive 60%	Quantitative
Chou et al. (2017) [31]	Taiwan	321 CRC survivors aged 62.0 ± 11.5 years, with 46.4% female	Stage I–IV ≥ 3 months since treatment (mean 21.6; range 3–60 months)	Face-to-face interviews for assessing the survey	Exercise—150 min a week	Ecological model of health behaviour	Quantitative descriptive 60%	Quantitative
D’Andrea et al. (2014) [30]	USA	2378 CRC survivors, with highest proportion aged 65 + years, with about the same ratio of males/females (not specified)	Stage and time since diagnosis were not specified	Personal household interviews for quantitative data	Leisure time physical activity	-	Quantitative descriptive 60%	Quantitative
Kim et al. (2021) [29]	South Korea	242 CRC survivors aged 54.1 years, with 47.5% female	Stage I–IV < 1 to ≥ 2 Years after treatment	Descriptive survey	Physical activity	Self Determination theory	Quantitative descriptive 80%	Quantitative
Lynch et al. (2010) [28]	Australia	403 CRC survivors, highest proportion of 42.7% aged 70 + years, with 38.5% female	Stage Dukes’ A–D ‘Time 2: 12 months’ since diagnosis was used	Computer-assisted telephone interviews for quantitative data	Physical activity	Ecological model of health behaviour	Quantitative descriptive 40%	Quantitative
Lynch et al. (2016) [27]	Australia and Canada	185 CRC colon cancer survivors aged 64.2 ± 10.3 years with 44.9% female	Stage I–IV 18.8 ± 4.4 months since diagnosis	Accelerometer-based assessments and survey	Physical activity (the Moderate to vigorous physical activity data was used) and sedentary time	-	Quantitative descriptive 60%	Quantitative
Packel et al. (2015) [26]	USA	96 CRC survivors aged 65.6 ± 11.7 years. Sex of participants was not specified	Stage 0–IV > 6 months out of active treatment	Cross-sectional quantitative survey	Physical activity behaviours	Theory of Planned Behaviour	Quantitative descriptive 60%	Quantitative
Peddle et al. (2008) [25]	Canada	413 CRC survivors aged 60 ± 7.5 years, with 46% female	Stage of disease was not specified Completed adjuvant therapy for at least 1 year	Cross-sectional mailed survey	Exercise behaviour	Self Determination Theory, psychological needs satisfaction in exercise (PNSE), perceived autonomy support (PAS)	Quantitative descriptive 80%	Quantitative
Van Putten et al. (2016) [24]	Netherlands	2451 CRC survivors aged 69.6 ± 9.5 years, with 45% female	Stage I–III 5.3 ± 2.8 years since diagnosis	Validated questionnaires	Moderate to vigorous physical activity	Theory of Planned Behaviour, Health Belief Model	Quantitative descriptive 60%	Quantitative

^**†**^Percentage is an indication for the quality of a paper: a high percentage is high-quality; lower percentage is a lower quality. This is assessed with the Mixed Method Appraisal tool (MMAT version 11, 2018) [22]. Five questions for each study were answered with either ‘Yes’, meaning criteria is met, ‘No’, meaning criteria is not met; or ‘Can’t tell’, meaning information is unconvincing to judge this criterion. Here, we display how much percent of the questions were answered with ‘Yes.’ Two studies scored a ‘Can’t tell’ on one of the questions, and these questions were scored with no points [30, 31]. One study was a mixed-method study, but results were used from the qualitative part only; therefore, the quality of the study assessed reflects the qualitative part of the study only [40]

^**‡**^Two studies were classified as ‘quantitative descriptive studies’, but reported, next to associations, also percentages of some of the assessed determinants and therefore those were mentioned in the qualitative results and the association in the quantitative results [34, 35]. One of the quantitative studies showed results of a survey and only reported frequencies and no associations of determinants with exercise and was therefore analysed with the qualitative studies [33]

### Behavioural determinants: quantitative descriptive studies

Twelve out of 21 studies assessed determinants with quantitative methods (see Table 2) [24–35]. Eleven of these studies assessed determinants for physical activity [24–33, 35] and one study looked at dietary changes [34]. However, one of the quantitative studies showed results of a survey and only reported frequencies and no associations of determinants with exercise and was therefore analysed with the qualitative studies [33]. In total, 61 determinants were assessed, of which roughly 80% were classified as having unconvincing evidence due to limited studies available or conflicting results (Table 3). The determinants that were assessed the most were age, sex, marital status, stage of disease and weight/BMI. Socio-demographic and clinical factors were analysed by nine [24–27, 29–32, 34] and ten studies [24–27, 29–32, 34, 35], respectively, but the results were mixed. There was convincing evidence that the following socio-demographic and clinical factors were ***not*** determinants of health behaviours: sex, socio-economic status, health insurance, comorbidities, stage of disease, type of cancer (i.e. colon or rectal), time since diagnosis and type of treatment. Seven studies assessed lifestyle factors [24, 26, 27, 30–32, 34], and only current smoking seems to be a determinant of healthy lifestyle behaviours as there is convincing evidence that this is negatively associated with other healthy lifestyle behaviours. Six studies assessed intrapersonal factors [25, 26, 28, 29, 31, 35]. The only convincing intrapersonal determinant of healthy lifestyle behaviours was ‘Lack of time and other priorities’, which seems to be associated with less healthy lifestyle behaviours. Factors in the category social environment and interpersonal factors were only assessed by two studies [28, 35] and had unconvincing evidence for being associated with healthy lifestyle behaviours. Only three studies assessed factors related to well-being [24, 29, 31]. From these studies, there was convincing evidence that having a depression is associated with less healthy lifestyle behaviours. Seven studies assessed symptom-related barriers [24, 26, 28, 31, 32, 34, 35]. The most researched symptom was cancer-related fatigue, but the results were inconclusive regarding the relation with health behaviour as three studies saw a negative association of cancer-related fatigue with healthy lifestyle behaviours [24, 32, 35], while two other studies found no association [26, 31]. There is convincing evidence that experiencing pain is negatively associated with healthy lifestyle behaviours and that having a good functional status (i.e. a person’s ability to perform everyday tasks across different domains, such as the social, cognitive and emotional domain) was positively associated with health behaviours. Only three studies assessed environmental factors [28, 31, 35], but there were too few studies that assessed each factor, and results were consequently classified as ‘unconvincing evidence’.

**Table 3:** Determinants of healthy lifestyle behaviours in quantitative studies (*n* = 11)

Determinants	Studies (*n*)^†^	Factors *positively* influencing healthy lifestyle behaviours	Factors *negatively* influencing healthy lifestyle behaviours	*No association* with healthy lifestyle behaviours	Level of evidence^‡^
Socio-demographic factors					
Age	8	55–74 years vs. ≥ 75 years [24]^§^; Higher age [26]	≥ 65 years [34]; higher age [27]	[24, 25, 30–32]	Unconvincing
Sex	7	Male [24]		[25, 26, 30–32, 34]	Convincing
Socio-economic status	5	Annual household income of ≥ $80.000 vs. < $40.000 [27]	Financial difficulties [24]	[29, 34]; poverty [30]; annual household income of $40.000–$79.000 vs. < $40.000 [27]	Convincing
Occupational status	4		Unemployed vs. part-time/full time [31]^¶^; not working vs. working [27]	[25–27]	Unconvincing
Education/years of education	5	Medium vs. low [24]; Some college education/ degree vs. < high school education [30]	Less education [25]	[24, 26, 31]	Unconvincing
Living alone/marital status	6	Having a partner [24]	Without partner [31]	[25, 26, 29, 30]	Unconvincing
Health insurance	2			[30, 32]	Convincing
Race/ethnicity	2	‘Other’ and more than one race [30]	Hispanics vs. non-Hispanic whites [30]	[26]	Unconvincing
Clinical factors					
Comorbidities	4		≥ 2 conditions [30]	[24, 31, 34]	Convincing
Stage of disease	6	Stage I, II [29]	II vs. I [24]	[24, 26, 29, 31, 32, 34]	Convincing
Type of cancer: colon or rectal	4			[24–26, 34]	Convincing
Years/months since diagnosis	5			[24, 25, 27, 32, 34]	Convincing
Treatment (chemotherapy, surgery, hormone and/or radiation)	4			[24–26, 34]	Convincing
Period after completing treatments	1			[31]	Unconvincing
Went back on cancer treatment	1		[35]		Unconvincing
Recurrence (local and metastatic)	2		[35]	[25]	Unconvincing
Lifestyle factors					
Current dietary regimen	1	[34]			Unconvincing
Received dietary advice	1	[34]			Unconvincing
Weight/BMI	7	Normal weight vs. obesity and overweight vs. obesity [24]	Obese vs. normal weight [27, 32]	[26, 30, 31, 34]	Unconvincing
Adherence to PA norm	1			[34]	Unconvincing
Current smoking	3		[30, 32, 34]		Convincing
Current alcohol use	2	[30]		[34]	Unconvincing
Intrapersonal factors					
Beliefs of importance	1		[31]		Unconvincing
Perceived outcomes	1	Feel better and improve wellbeing, reduce the risk of cancer returning, relieve stress, improve energy level, get mind of cancer, live longer, improve fitness, lose weight, improve immune system [35]			Unconvincing
Level of being interested/attitude	2		[31]	[26]	Unconvincing
Motivation	2	Introjected regulation [25]; Identified regulation [25]		Autonomous [29]; Amotivation, external and intrinsic regulation [25]	Unconvincing
Competence	2	[29]		[25]	Unconvincing
Relatedness	2	[29]		[25]	Unconvincing
Perceived autonomy support	1			[25]	Unconvincing
Need for autonomy	1			[25]	Unconvincing
Intention	1			[26]	Unconvincing
Perceived behavioural control	1	[26]			Unconvincing
Lack of time and other priorities	2		[31, 35] Lack of time and additional family responsibilities [35]		Convincing
Pleasure and fun	1		Boring activity [35]		Unconvincing
Personal attributes	1			Fear of injury self, lack of enjoyment or interest in PA, have never been physically active, already active enough [28]	Unconvincing
Social environment and interpersonal factors					
Social influence	1	Spouse/partner, other family members, best friend, oncologist [35]			Unconvincing
Social environmental barriers	1			Lack of time, too many other commitments, no encouragement from family or friends, no encouragement from doctor [28]	Unconvincing
Mental/ well-being factors					
Anxiety	2		[24]	[31]	Unconvincing
Depression	3		[24, 29, 31]		Convincing
Global quality of life	1	[24]			Unconvincing
Body image distress	1			[24]	Unconvincing
Future perspective	1	[24]			Unconvincing
Disease-related symptoms					
Fatigue	5		[24, 32, 35]	[26, 31]	Unconvincing
Stoma (yes)	4		[24, 34]	[26]; stoma-related problems [24]; barriers: concerns about leakage from pouch, pouch is uncomfortable and makes it hard to exercise [28]	Unconvincing
Disease-specific barriers	1		Fatigue, diarrhoea, or incontinence, do not feel well enough to be physically active [28]		Unconvincing
Insomnia	2		[24]	[31]	Unconvincing
Pain	3		[24, 31, 35]		Convincing
Dyspnoea	1		[24]		Unconvincing
Neuropathy	1			[26]	Unconvincing
Appetite loss	1		[24]		Unconvincing
Micturition problems	1		[24]		Unconvincing
Chemo-side effects	1		[24]		Unconvincing
Gastro-intestinal problems	1			[24]	Unconvincing
Defecation problems	1			[24]	Unconvincing
Weight loss	1		[24]		Unconvincing
Nausea	1			[32]	Unconvincing
Somatization	1		[32]		Unconvincing
Medical/health problems	1		[35]		Unconvincing
(Good) functional status	2	[31]	Barrier: Physical, Role, Social, Emotional and Cognitive functioning [24]		Convincing
Environmental factors					
Bad weather	2	[31]	[35]		Unconvincing
Physical environmental barriers	1			Lack of suitable facilities, locale perceived as unsafe, locale perceived as unattractive [28]	Unconvincing

**†**All studies examined determinants for physical activity or exercise, apart from one study that looked at dietary changes [34]

**‡** In line with a comparable systematic review on determinants of exercise in cancer survivors [18], the level of evidence was classified as ‘convincing’ when there were consistent findings in ≥ 2 high-quality studies, ‘moderately convincing’ when findings were consistent in one high-quality study and at least one low-quality study or where consistent in ≥ 2 low-quality studies and ‘unconvincing’ when there was only one study available or findings were inconsistent in ≥ 2 studies. Results were consistent when ≥ 75% of the studies showed results in the same direction [18]

**§** After consultation with the authors of this study, the data from the tables was used due to inconsistencies between the tables and the text

**¶** In this study, the following barriers were reported: Lack of time (31.5%), bad weather (58.6%), without partner (2.8%), lack of energy (12.1%), no suitable place (2.2%), but only the first three barriers were analysed further [31]

### Behavioural determinants: qualitative studies

Twelve out of 21 studies assessed determinants in a qualitative manner [33–44], of which two studies also included quantitative analyses and were partly analysed with the quantitative studies [34, 35] (see Table 2). Three studies focused on dietary behaviours [34, 36, 38], six on exercise and/or physical activity [33, 35, 37, 39–41], and three studies on both dietary behaviours and exercise or physical activity [42–44]. Twenty-one different determinants could be distinguished (Table 4). Only one socio-demographic factor was assessed [41], and the evidence was unconvincing. No clinical and lifestyle factors were assessed. All 12 studies examined intrapersonal factors. The following intrapersonal factors had convincing evidence for being determinants of healthy lifestyle behaviours: (lack of) motivation, (lack of) knowledge, perceived and expected outcomes, (lack of) time and other priorities, (false) beliefs and (lack of) skills. These factors could be both barriers and facilitators for adhering to healthy lifestyle behaviours, depending on whether people score low or high on them. Eight studies examined determinants in the category social environment and interpersonal factors [33, 35–39, 41, 43]. Both (lack of) social support and (lack of) social norms and influence were determinants of healthy lifestyle behaviours and could be both barriers and facilitators for healthy lifestyle behaviours. Factors related to (mental) well-being were only examined by three studies [33, 39, 42]. Only for ‘body image distress/consciousness’, there was convincing evidence that this was associated with less healthy lifestyle behaviours. Factors in the category disease-related symptoms were assessed by seven studies [33, 35, 37, 39, 40, 43, 44]. Determinants with convincing evidence for being a determinant of healthy lifestyle behaviours were dealing with symptoms and having a bad health status (i.e., being in a poor health condition for example due to illness or injury). These factors seem to be associated with less healthy lifestyle behaviours. The two assessed environmental factors ‘weather’ and ‘(lack of) access to facilities and equipment’ had convincing evidence for being determinants of healthy lifestyle behaviours. These factors were examined by five studies [33, 35, 39–41] and could be both barriers and facilitators for adhering to healthy lifestyle behaviours.

**Table 4:** Determinants of health behaviours in qualitative studies (*n* = 9) and quantitative studies who presented frequencies from surveys (*n* = 3)

Categories	Determinants	Studies (*n*)^†^	Sub-determinants and description	Ref.^‡^	Level of evidence^§^
Socio-demographic factors	Age and energy	1	Age and energy	[41]	Unconvincing
Intrapersonal factors	(Lack of) motivation	8	Motivators: Prevent recurrence, support therapy and recovery, reduce cancer-related complaints, other: promote bowel movements, lose weight, and improve general health Enjoyment, health benefits (mental and physical), sense of achievement, weight management, sense of normalcy, spending time on themselves away from daily responsibilities Lack of motivation to change Motivators: exercising with others, doing a specific activity, seeing benefits/results, adding entertainment, competition, doing a variety of activities/sports Too much effort/lack of willpower Lack of motivation Lack of discipline or willpower and lack of interest Motivation but not sure how: Intentions, ‘It’s hard for me, but I’m trying to change’	[34] [39] [44] [35] [41] [40] [33] [42]	Convincing
	(Lack of) knowledge	7	Insufficient knowledge of guidelines, guidelines not applicable, doing sufficient physical activity Lack of knowledge and conflicting information Desire for health information: ‘You can’t do what you don’t know’ Increase in knowledge by receiving information from health care professionals Importance of correct information of specialist and need for detailed exercise information Lack of knowledge on how to do exercise Unclear guidance	[41] [43] [42] [36] [37] [33] [39]	Convincing
	Perceived and expected outcomes	7	Benefits to exercise: health, weight, diet, reduce fatigue, cardiovascular health, unsure Benefits to physical activity: weight control, improve physical health, improve physical fitness, improve cardiovascular health, improve mental health, improve physical function, build strength, and feel better/healthier Beliefs regarding feeling better physically, beliefs regarding feeling better psychologically, I do not want to get sick again, expectation for health and fitness improvements, expectation for improved bowel function and expectation for better defecation Awareness of the importance of healthy diet after treatment and benefits of dietary changes such as general well-being Fear of injury Fear of injury and negative past experiences Facilitate recovery, manage treatment side effects, avoid disruption of treatment, and prevent food drug interaction	[40] [35] [37] [36] [33] [39] [38]	Convincing
	(Lack of) time and other priorities	5	Having time Competing priorities/lack of time Time as barrier Time constraints Lack of time, work demands, family demands and social demands	[35] [41] [40] [37] [33]	Convincing
	(False) beliefs	4	The pleasures in life: is it worth it? and beliefs about health behaviours Beliefs in divine control: Fatalism (i.e. beliefs about whether cancer outcomes can be changed) and Religion/spirituality; Personal responsibility: ‘You’re supposed to help yourself’ Individual commitment to dietary change; facilitators: traditional Chinese beliefs, barriers: traditional cultural beliefs and practices Trial and error approach, traditional Chinese remedies and Illness causal beliefs (protections from future recurrence, perceptions of former diet, conformance to social norms)	[44] [42] [36] [38]	Convincing
	(Lack of) skills	4	Need for simple messages and strategies to stay healthy Lack of skills Skills Coping skills: Adaptive strategies in interpersonal contexts: avoid feeling isolated while eating with others and challenges during Chinese festivals and innovative strategies to overcome these challenges	[43] [33] [39] [36]	Convincing
	Attitude	1	Scepticism of eating guidelines	[44]	Unconvincing
	Identity	1	Not the sporty type	[41]	Unconvincing
	Self-efficacy	1	Confidence	[39]	Unconvincing
	Pleasure and fun	1	Does not enjoy exercise, exercise is hard work, and boring	[33]	Unconvincing
Social environment and interpersonal factors	(Lack of) social support	6	Companionship Lead and interaction with an exercise specialist, exercising with other CRC survivors, exercising with others (in general), and solo exercise is less fun Social support, medical surveillance and insufficient physical activity advice from medical professionals No exercise buddy Desire for support Social support and support networks, and guidance	[35] [37] [41] [33] [43] [39]	Convincing
	(Lack of) social norms and influence	4	Normative approve: family, spouse/partner, friends, healthcare professional, children, grandchildren; Normative disapprove: friends, healthcare professional, spouse, family, parents, siblings Normative beliefs: oncologist, family and friends, spouse, and other CRC survivors Working with healthcare professionals during the journey Family influence and cultural ‘sick role’ beliefs	[35] [37] [36] [38]	Convincing
(Mental) well-being factors	Body image distress/consciousness	2	Self-consciousness about looks Public and private self-consciousness and uncertainty in unfamiliar surroundings	[33] [39]	Convincing
	Resilience	1	Resilience	[42]	Unconvincing
Disease-related symptoms	Dealing with symptoms	5	Bowel changes Back to normal Injuries and side effects of cancer treatment Cancer treatment/recovery Negative side effects of cancer and treatments, physical restrictions, stoma, and experimentation: to minimize stoma-related complaints, past experiences with dealing with a stoma while being physically active	[43] [44] [37] [35] [39]	Convincing
	Bad health status	3	Being healthier as facilitator, poor health/body condition, musculoskeletal, arthritis, and deconditioned Illness and injury Too overweight and poor health	[35] [40] [33]	Convincing
	Pain	1	Minor aches and pain	[33]	Unconvincing
	Fatigue	1	Lack of energy and too tired	[33]	Unconvincing
Environmental factors	Weather	4	Weather as barrier Good weather Hot weather Bad weather	[40] [35] [41] [33]	Convincing
	(Lack of) access to facilities and equipment	4	Barriers: transportation, location, and money Facilitators: environment (e.g. proximity to facility, somewhere to walk) Lack of equipment, lack of money, lack of convenient place Safe environment	[40] [35] [33] [39]	Convincing

**†**Three studies were quantitative studies who presented frequencies from surveys [33–35]

**‡***Ref.*, Reference

**§**In line with a comparable systematic review on determinants of exercise in cancer survivors [18], the level of evidence was classified as ‘convincing’ when there were consistent findings in ≥ 2 high-quality studies, ‘moderately convincing’ when findings were consistent in one high-quality study and at least one low-quality study or where consistent in ≥ 2 low-quality studies and ‘unconvincing’ when there was only one study available or findings were inconsistent in ≥ 2 studies. Results were consistent when ≥ 75% of the studies showed results in the same direction [18]

### Summary of identified determinants of lifestyle behaviours

**Fig. 2:**
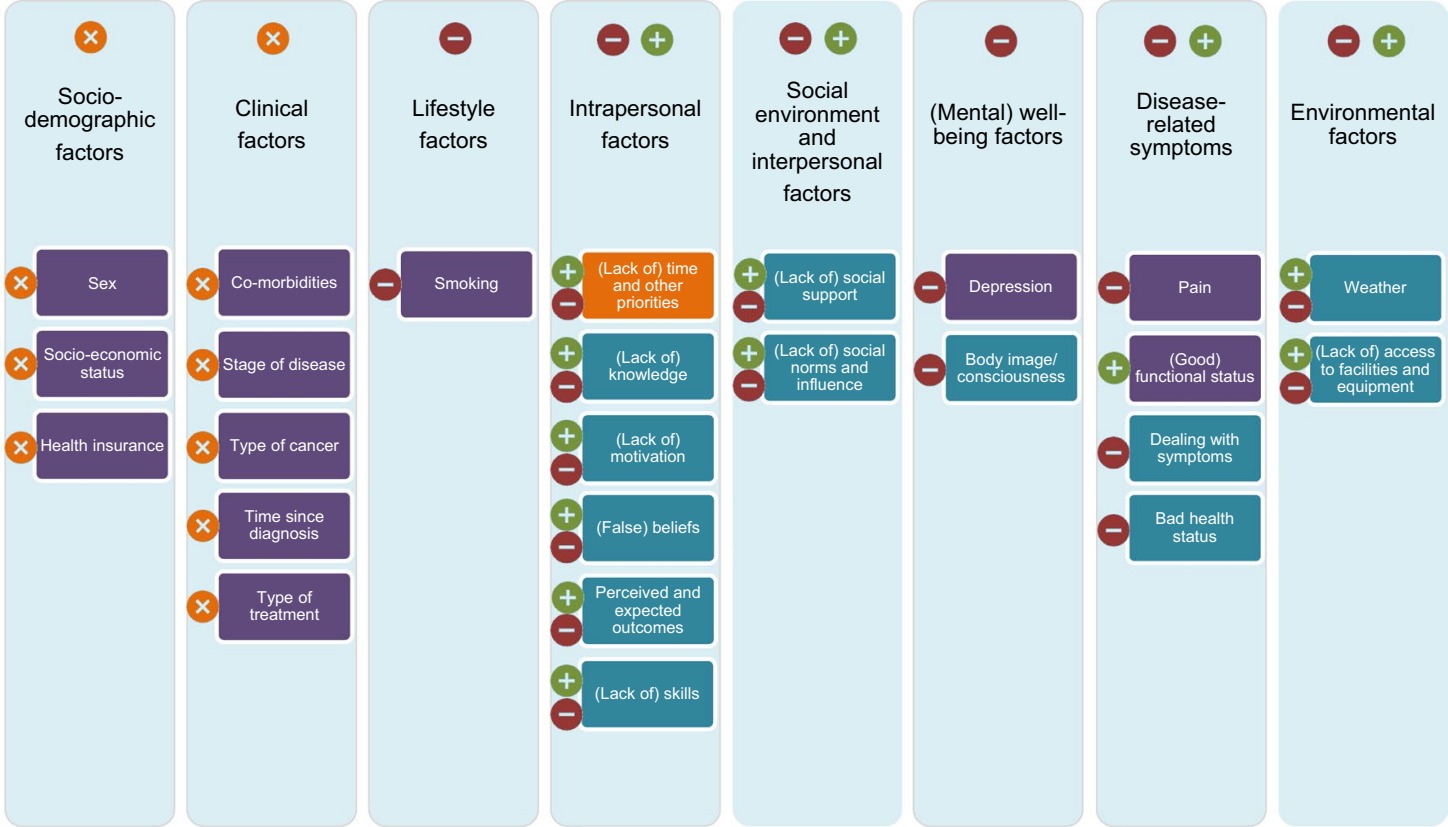
Summary of convincing evidence for determinants of healthy lifestyle behaviours from quantitative studies (in purple), qualitative studies (in aqua) or both type of studies (in orange) (*n*=21). The direction of the determinant is indicated: x = evidence for no association, - = evidence for a negative association, and + = evidence for a positive association

Figure 2 summarizes the identified determinants that had convincing evidence either in quantitative, qualitative studies or both, for being determinants of healthy lifestyle behaviours in CRC survivors who completed treatment. The determinants smoking, depression, body image distress/consciousness, pain, dealing with symptoms and bad health status were associated with less healthy lifestyle behaviours. (Good) Functional status was associated with more healthy lifestyle behaviours. The determinants (lack of) time and other priorities, (lack of) knowledge, (lack of) motivation, (false) beliefs, perceived and expected outcomes, (lack of) skills, (lack of) social support, (lack of) social norms and influence, weather and (lack of) access to facilities and equipment could either be associated with less or more healthy lifestyle behaviours depending on whether people score low or high on them. The following factors do ***not*** seem to be important determinants of lifestyle behaviours: sex, socio-economic status, health insurance, comorbidities, stage of disease, type of cancer (i.e. colon or rectal), time since diagnosis and type of treatment.

## Discussion

This systematic review aimed to identify determinants of healthy eating, physical activity and/or exercise in CRC survivors contributing to a better understanding of which determinants should be addressed to promote healthy lifestyle behaviours in this population. Intrapersonal determinants with convincing evidence (i.e. consistent findings in ≥ 2 high-quality studies) for an association with less or more healthy lifestyle behaviours were time and other priorities, knowledge, motivation, (false) beliefs, perceived and expected outcomes and skills. The identified determinants in the categories ‘social environment and interpersonal factors’ (i.e. social support, social norms and influence) and ‘environmental factors’ (i.e. access to facilities and equipment, and weather) also seem to be associated with either less or more adherence to healthy lifestyle behaviours. Disease-related symptoms were generally associated with less healthy lifestyle behaviours, although good functional status seems to be associated with more healthy lifestyle behaviours. (Mental) Well-being factors were not frequently assessed. However, the studies that did assess the (mental) well-being factors depression and body image distress/consciousness seem to suggest an association with less healthy lifestyle behaviours. Lifestyle factors, such as weight/BMI and alcohol use, were rarely assessed by the included studies, except for smoking which was negatively associated with healthy lifestyle behaviours. Socio-demographic and clinical factors were either not associated with healthy lifestyle behaviours or there was unconvincing evidence (i.e. only one study available, or findings were inconsistent in ≥ 2 studies).

Another systematic review that focussed on factors that influenced healthy eating behaviours (and not physical activity) among CRC survivors identified factors related to outcome expectancies (i.e. expected costs and benefits of healthy behaviours), knowledge and social surroundings, which aligns with the results of our review [16]. There were also some differences in our findings compared to theirs, as they did not find motivation, body image distress/consciousness, the lack of time and other priorities and weather to be important determinants of healthy eating behaviours. These differences may arise from the other review’s exclusive focus on eating behaviours, with no clear indication of the survivorship stage under consideration. Body image distress typically manifests as a symptom post-treatment, which is the focus of the current review, for example, when a stoma has been placed [11]. The role of time and weather may be more important determinants for physical activity than eating behaviours, as all studies in our review that identified these determinants examined exercise or physical activity behaviours [31, 33, 35, 37, 40, 41].

The results of our review seem to indicate that lifestyle interventions for CRC survivors should prioritize targeting changeable determinants of health behaviour, such as motivation and social support, over fixed factors such as clinical factors. Additionally, our findings suggest that intrapersonal factors, disease-related symptoms and (mental) well-being factors are associated with healthy lifestyle behaviours. What stands out for this population is that CRC survivors are typically dealing with gastro-intestinal problems, bowel changes and a stoma. From the included qualitative studies, we, for example, inferred that CRC survivors could be more motivated to change healthy lifestyle behaviours when they expected it to improve bowel function. Also, pain was an important barrier to lifestyle behaviours in our review. This might be more related to physical activity than eating behaviours and also more prominent after treatment, as the earlier discussed review on eating behaviours did not identify pain as a determinant [16] and the studies in our review that identified pain as determinant all examined exercise or physical activity behaviours [24, 31, 35]. We also identified (mental) well-being factors as important determinants of lifestyle behaviours. Taking mental wellbeing into account is important as prevalence of, for example, anxiety and depression in CRC survivors exceeds those of the normative population [45]. The importance of addressing (mental) wellbeing for changing lifestyle behaviours is supported by findings from another review on healthy eating behaviour in CRC survivors in any treatment stage [16] and a review on exercise in cancer survivors who completed treatment [17].

Complementary to tailoring interventions to intrapersonal factors, disease-related symptoms and (mental) well-being factors, interventions should also target a broad range of other underlying factors for lifestyle behaviour change, such as social and environmental factors [18, 46, 47]. According to the socio-ecological model, there are multiple influences on health behaviours across different levels, such as the intrapersonal and interpersonal level [47]. Interventions that address determinants on different levels should be most effective in sustaining a change in behaviour [47]. Social environment and interpersonal factors, such as social support, norms and influence, are important determinants of lifestyle behaviours as identified in our review and other reviews on healthy eating behaviour in CRC survivors in any treatment stage [16] and on exercise in cancer survivors who completed treatment [17]. For example, lacking someone to exercise with may serve as a barrier to exercise [17], while having a family member who eats healthy could influence the adoption of healthy eating behaviours [16]. An important environmental determinant that was identified in the current study was (lack of) access to facilities and equipment, which stresses the importance of addressing broader environmental factors in lifestyle interventions. Additionally, empowering individuals with effective coping strategies to overcome environmental barriers is important for promoting sustained behaviour change. Implementing strategies such as providing resources like sport facilities or facilitating community initiatives to enhance access to healthy food options can assist individuals in overcoming environmental challenges and maintaining healthy lifestyle behaviours [48]. Moreover, personalized approaches that consider individual preferences, resources and environmental barriers can enhance the effectiveness of behaviour change interventions.

Although the included quantitative papers showed unconvincing evidence for socio-economic status as a direct determinant of healthy lifestyle behaviours, the qualitative papers mentioned ‘lack of money’ as an important aspect within the determinant ‘(Lack of) access to facilities and equipment’. This also suggests the importance of considering the indirect effects of determinants on health behaviours. It could be speculated that CRC survivors are inclined to adopt healthy lifestyle behaviours post-diagnosis, regardless of their socio-economic status. However, the studies in our review that assessed socio-economic status are difficult to compare due to the use of different assessment methods for socio-economic status. To illustrate, one study assessed the annual house income [27], while another study assessed poverty [30]. In addition, two studies that reported no association between socio-economic status and healthy lifestyle behaviours, predominantly included participants with a medium to high socio-economic status [29, 34]. Furthermore, other reviews did identify socio-economic status as a determinant of healthy eating behaviour in CRC survivors [16], and costs were considered a barrier to exercise in cancer survivors [17]. Due to the limitations of the studies included in our review and the evidence from other reviews, we think that interventions can benefit from considering socio-economic status, especially available money, in lifestyle interventions for CRC survivors. This can be done by, for example, creating interventions that fit the needs and resources of groups of different socio-economic status. Lifestyle factors were rarely assessed. However, one can argue whether lifestyle factors, such as smoking and alcohol use, are actual determinants of lifestyle behaviours, or whether they have a bidirectional relationship with other lifestyle behaviours. Meaning that lifestyle behaviours can influence each other. To illustrate, smoking might lead to decreased physical activity and poor dietary choices, but individuals with unhealthy lifestyle behaviours and decreased physical activity might also be more likely to be smokers.

### Strengths and limitations

This study has three important strengths. First, as far as we know, this is the first time that an overview of determinants of healthy eating and/or physical activity or exercise is provided in CRC survivors who completed treatment. It is important to differentiate where CRC survivors are in their cancer journey and tailor interventions to the specific needs that match the different phases. CRC survivors who completed treatment deal with different side effects and possibly a long-term stoma and shift their focus from survival to dealing with the aftermath of the disease. Second, due to the sparsity of overviews of determinants for healthy lifestyle behaviours, particularly dietary habits, the challenge in designing trials is to identify which determinants to target among CRC survivors to encourage the adoption of healthy lifestyle behaviours. The current paper contributes to this choice-making and can prevent researchers from going through the extensive process of identifying these determinants for CRC survivors. Third, we examined both qualitative and quantitative studies. Qualitative and quantitative studies have different strengths that can complement each other to provide a more validated and robust analysis. Qualitative studies provide more in-depth analyses of individual’s perceptions, experiences and attitudes and explore contexts and can therefore enhance interpretation of statistical associations. Quantitative studies can provide more precise measurements and offer insights in generalizability of the data. The quantitative studies in this review often assessed a set of pre-listed determinants, and therefore, it is possible that certain determinants are not identified. The results from the qualitative studies are therefore a valuable addition, as these generated new determinants. For example, while socio-economic status was not convincingly identified as determinant in the quantitative studies, a specific aspect of socio-economic status, the lack of money, was a determinant under the category of ‘(Lack of) access to facilities and equipment’ in the qualitative studies.

There are three limitations to discuss. First, due to the sparsity of studies that focus on dietary behaviours, it was not possible to analyse the results separately for nutrition and physical activity or exercise. This may have influenced the comprehensiveness of our findings and the depth of our understanding regarding the distinct determinants of each behaviour. Second, the included studies used ambiguous terms for the determinants, which makes it difficult to determine the similarity of the determinants across studies and to cluster them into determinant groups. This theoretical heterogeneity in behaviour change theories is a commonly encountered phenomenon that hinders the replication and integration of results [49, 50]. In addition, some of the studies we included discussed general barriers and/or facilitators. The grouping of determinants as either barrier or facilitator might be an oversimplification of the complexity of behaviour change as some determinants, such as time, knowledge and facilities might both have a supportive and inhibitory role in changing behaviour. Therefore, in our result synthesis, we divided the determinants that were listed as barriers and facilitators over the determinant groups. Third, to summarize the evidence, we considered factors as convincing determinants for healthy lifestyle behaviours, when they had convincing evidence in either the quantitative studies or qualitative studies. It would be stronger to find convincing evidence for a determinant in both study types. However, the quantitative studies often assessed different factors than the qualitative studies, which made it impossible to make this comparison. Quantitative studies mostly focused on socio-demographic and clinical factors, whereas the qualitative studies mostly focussed on intrapersonal factors. While quantitative and qualitative studies can complement each other in identifying different determinants, future research should adopt a more holistic approach and consider a broader set of variables to validate determinants across study types.

Moving forward, it is important to not only consider determinants as independent factors but also look at determinants in interaction, as interactions between determinants across different levels are highly likely [47]. These interactions may influence behaviour change in combined or synergistic ways, for example, when the expected benefits of physical activity increase motivation for starting or continuing physical activity. Understanding these interactions can contribute to a more comprehensive understanding of the complexity of behaviour change and can aid in the development of interventions. To effectively change the identified determinants in this review, they should be coupled with behaviour change techniques [51–53]. Moreover, we recommend to tailor interventions to the individual’s needs and wishes when changing complex behaviour. For example, while some CRC survivors may need to increase their knowledge of healthy eating behaviours, others may not, and not all CRC survivors have a stoma, necessitating tailored interventions. By adopting a personalized approach, interventions can better address the diverse needs of individuals, which helps to enhance their effectiveness in promoting behaviour change [46].

## Conclusion

Our review highlights determinants for healthy lifestyle behaviours among CRC survivors who completed treatment. Intrapersonal factors, social environment and interpersonal factors, (mental) well-being factors, disease-related symptoms and environmental factors seem especially important for this population. Interventions for changing health behaviours in CRC survivors who finished treatment could use these determinants to tailor and personalize the intervention to the target group.

## Supplementary Information

Below is the link to the electronic supplementary material.

Supplementary file1 (DOCX 35 KB)

## Data Availability

No datasets were generated or analysed during the current study.
