# Supplementary material for: Determinants of healthy lifestyle behaviours in colorectal cancer survivors: a systematic review
Source: Support Care Cancer. 2025 Mar 18;33(4):292. doi: 10.1007/s00520-025-09315-x (PMC11914313; doi:10.1007/s00520-025-09315-x)
Supplement: Supplementary file 1 — Supplementary file1 (DOCX 35 KB) [file 520_2025_9315_MOESM1_ESM.docx]

Supporting information: Quality assessment

*Table S1. Quality analysis of quantitative studies*

| Study | S1. Are there clear research questions? | S2. Do the collected data allow to address the research questions? | 4.1. Is the sampling strategy relevant to address the research question? | 4.2. Is the sample representative of the target population? | 4.3. Are the measurements appropriate? | 4.4. Is the risk of nonresponse bias low? | 4.5. Is the statistical analysis appropriate to answer the research question? |
| --- | --- | --- | --- | --- | --- | --- | --- |
| Bours et al. 2015 | Yes | Yes | Yes | No | Yes | Yes | Yes |
| Chambers et al. 2009 | Yes | Yes | Yes | No | Yes | No | Yes |
| Chou et al. 2017 | Yes | Yes | Yes | No | Yes | Can’t tell | Yes |
| D’Andrea et al. 2014 | Yes | Yes | Yes | Yes | No | Can’t tell | Yes |
| Kim et al. 2021 | Yes | Yes | Yes | No | Yes | Yes | Yes |
| Liu et al. 2023 | Yes | Yes | Yes | No | Yes | Yes | Yes |
| Lynch et al. 2010 | Yes | Yes | No | Yes | No | No | Yes |
| Lynch et al. 2016 | Yes | Yes | Yes | No | Yes | No | Yes |
| Packel et al. 2008 | Yes | Yes | Yes | No | Yes | No | Yes |
| Peddle et al. 2008 | Yes | Yes | Yes | Yes | Yes | No | Yes |
| Speed-Andrews et al. 2014 | Yes | Yes | Yes | Yes | Yes | No | Yes |
| Van Putten et al. 2016 | Yes | Yes | Yes | No | Yes | No | Yes |

*Table S2. Quality analysis of qualitative studies*

| Study | S1. Are there clear research questions? | S2. Do the collected data allow to address the research questions? | 1.1. Is the qualitative approach appropriate to answer the research question? | 1.2. Are the qualitative data collection methods adequate to address the research question? | 1.3. Are the findings adequately derived from the data? | 1.4. Is the interpretation of results sufficiently substantiated by data? | 1.5. Is there coherence between qualitative data sources, collection, analysis and interpretation? |
| --- | --- | --- | --- | --- | --- | --- | --- |
| Byeon et al. 2024 | Yes | Yes | Yes | Yes | Yes | Yes | Yes |
| Hardcastle et al. 2017 | Yes | Yes | Yes | Yes | Yes | Yes | Yes |
| Hardcastle et al. 2018 | Yes | Yes | Yes | Yes | Yes | Yes | Yes |
| Harper et al. 2013 | Yes | Yes | Yes | Yes | Yes | Yes | Yes |
| Maxwell-Smith et al. 2017 | Yes | Yes | Yes | Yes | Yes | Yes | Yes |
| Ray et al. 2018 | Yes | Yes | Yes | Yes | Yes | Yes | Yes |
| Saunders et al. 2019 | Yes | Yes | Yes | Yes | Yes | Yes | No |
| Tang et al. 2019 | Yes | Yes | Yes | Yes | Yes | Yes | Yes |
| Wong et al. 2021 | Yes | Yes | Yes | Yes | Yes | Yes | Yes |
